# Pattern of vitreo-retinal diseases at the national referral hospital in Bhutan: a retrospective, hospital-based study

**DOI:** 10.1186/s12886-020-01335-x

**Published:** 2020-02-13

**Authors:** Bhim B. Rai, Michael G. Morley, Paul S. Bernstein, Ted Maddess

**Affiliations:** 1 John Curtin School of Medical Research, Australian National University, Canberra, ACT 2601 Australia; 2 Department of Ophthalmology, JDW National Referral Hospital, Thimphu, Bhutan; 3 Ophthalmic Consultants of Boston, Harvard Medical School, Boston, MA USA; 4 Moran Eye Centre, University of Utah, Salt Lake City, UT USA

**Keywords:** AMD, Blindness, Diabetic macular oedema, Diabetic retinopathy, Pattern and presentation, Retinal diseases, SHAPU

## Abstract

**Background:**

Knowing the pattern and presentation of the diseases is critical for management strategies. To inform eye-care policy we quantified the pattern of vitreo-retinal (VR) diseases presenting at the national referral hospital in Bhutan.

**Methods:**

We reviewed all new patients over three years from the retinal clinic of the Jigme Dorji Wangchuck National Referral Hospital. Demographic data, presenting complaints and duration, treatment history, associated systemic diseases, diagnostic procedures performed, and final diagnoses were quantified. Comparisons of the expected and observed frequency of gender used Chi-squared tests. We applied a sampling with replacement based bootstrap analysis (10,000 cycles) to estimate the population means and the standard errors of the means and standard error of the 10th, 25th, 50th, 75th and 90th percentiles of the ages of the males and females within 20-year cohorts. We then applied t-tests employing the estimated means and standard errors. The 2913 subjects insured that the bootstrap estimates were statistically conservative.

**Results:**

The 2913 new cases were aged 47.2 ± 21.8 years. 1544 (53.0%) were males. Housewives (953, 32.7%) and farmers (648, 22.2%) were the commonest occupations. Poor vision (41.9%), screening for diabetic and hypertensive retinopathy (13.1%), referral (9.7%), sudden vision loss (9.3%), and trauma (8.0%) were the commonest presenting symptoms. Coexistent diabetes and hypertension were the most common associated systemic diseases. Haematological tests (blood sugar, HbA1c and lipid profile, 31.8%), OCT (27.4%), refraction (9.9%), B-scan (8.7%), fundus photography (8.0%) were the most commonly performed diagnostic tests. Hypertensive retinopathy (18.9%) was the commonest VR disease, followed by refractive errors referred for retinal evaluation (16.7%), diabetic retinopathy with macular oedema (15.8%), and AMD (11.0%). Retinal detachment was more prevalent in females (83 vs. 41, *p* = 0.007). Rare vision-threatening diseases like seasonal hyper-acute pan-uveitis also presented.

**Conclusions:**

The developing VR service in Bhutan is challenged by the spectrum of diseases, limited human resources (e.g. one retinal surgeon during the study), and accessibility to tertiary eye-care services, all amidst difficult terrain. Sustained effort and robust coordination among the eye-care professionals, government and non-governmental organisations are critical for optimising VR services, especially as rates of diseases such as diabetes and hypertension grow.

## Background

Bhutan has 73% of its land under forest cover and its terrain can be challenging. At the same time legislation does not permit the import of used vehicles or machinery, so aspects of Bhutan’s infrastructure can be modern. Taken together Bhutan’s fairly unique aspects have implications for the health of its population of 735,553 [1]. Here we concentrate on VR diseases.

Traditional healing methods were predominant in Bhutan until the 1960s [2]. A hierarchical system of healthcare centres is now established in the country: basic health units (BHU) equipped with X-Ray facilities at the village or block level, district hospitals at district level, and regional referral hospitals in each of the eastern, central and western regions. At each level there is emphasis on health advocacy to the public and collaboration among departments [2]. The first national ophthalmologist, Dr. Kunzang Getshen joined the civil service in 1987 and is honoured as the *Father of Ophthalmology* in Bhutan. Eye-care services improved rapidly under his leadership.

The Rapid Assessment of Avoidable Blindness (RAAB) survey conducted in 2009 and 2018 respectively reported 22.1% [3] and 7.7% [4] of visual impairment and blindness in Bhutan was due to posterior segment pathologies among the population aged ≥50 years. There has, however, never been a study conducted on the pattern of VR diseases in Bhutan. Within the region, a population-based study in India reported the overall prevalence of VR disorders to be 8.56% among the population aged 40 years and above [5]. The 1981 Nepal Blindness Survey reported VR disorders as the third leading cause of bilateral blindness, second only to cataract and its complications [6]. A population-based study in Nepal in 2013 reported VR disorders to be the second commonest cause of bilateral blindness, second only to cataract, and the most common cause among pseudophakics [7]. A more recent population-based study in Nepal reported that retinal disorders as the second most common cause of bilateral low vision, and the most common cause for blindness [8]. A RAAB survey in Bangladesh reported VR disease as the second leading cause of bilateral blindness [9].

Knowing the disease pattern and prevalence is important for planning national strategies and formulating action plans to control blindness effectively. The current study of 2913 cases was conducted to better understand the pattern and presentation of VR diseases presenting at the apex national referral hospital, and to provide baseline data for formulating action plans to control VR diseases in the country. The study has implications for other developing countries.

## Methods

### Study design

This was a retrospective cross-sectional case series study; approved by the Research Ethics Board of Health (REBH), Ministry of Health, Royal Government of Bhutan, Thimphu, Bhutan, and adhered to the principle of Declaration of Helsinki. The consent was waived by REBH because this retrospective study collected only the de-identified data.

### Setting

The Jigme Dorji Wangchuck National Referral Hospital (JDWNRH) is the apex national referral hospital located in Thimphu, the capital city. It provides subspeciality services in Cornea and anterior segment, Paediatric ophthalmology and strabismus, Glaucoma, Oculoplasty, Vitreo-retina. Currently, every BHU and district hospital provides primary eye-care services administered by ophthalmic technicians or optometrists. The regional referral hospitals are manned by teams of ophthalmologists, optometrists and ophthalmic nurses. The VR services in the country are provided only at the JDWNRH. All the VR patients across the country are referred to JDWNRH for management. Therefore, the pattern and presentation of the VR diseases at this hospital represents the VR diseases from the entire citizenry. JDWNRH also serves as the main clinical centre for the postgraduate medical students, interns and optometric and paramedic students. The current study was conducted at the VR Subspeciality Clinic (VRSC), Ophthalmology Department, JDWNRH, Thimphu, Bhutan.

### Participants

All new patients presenting to the VRSC over 3 years (01 May 2013 until 30 April 2016) were included in this study.

### Clinical examination and data collection

The data collected included the demographic information, presenting complaints and their duration, medical or surgical treatments received before presenting to the VRSC, and associated systemic diseases with their durations. Best corrected visual acuity (BCVA) at presentation was measured using a Snellen chart: Tumbling E for illiterate patients or the Sheridan Gardeners 3 m vision chart for children. Intraocular pressure was measured by Goldmann applanation tonometry or an iCare tonometer, and the anterior and posterior segments were examined under slit-lamp biomicroscopy (BM 900, Haag-Streit, Switzerland) and 90D bio-microscopy. The funduscopic findings were confirmed by binocular indirect ophthalmoscope (Model 125, Welch Allyn, USA). Macular and retinal nerve fibre (RNFL) scans were measured using a Spectral Domain OCT (Cirrus-HD 4000, Carl Zeiss Germany). Fundus photographs were taken by a VISUCAM-524 (Carl Zeiss, Germany). In case of children, the diagnoses were confirmed by examination under anaesthesia (EUA) if necessary, following a proper pre-anaesthetic check-up (PAC).

Hypertensive retinopathy (HTR) was graded using the Keith-Wagener-Barker classification system [10]. Diabetic retinopathy (DR) was classified as per the modified Airlie House or Abbreviated ETDRS classification [11]. Clinically significant macular oedema (CSMO) was defined as: 1) retinal oedema at or within 500 μm of the centre of the macula; 2) hard exudates at or within 500 μm of the centre of the macula if associated with retinal thickening (including outside 500 μm); 3) retinal thickening of one disc diameter (1500 μm) or larger, any part of which is within one disc diameter of the centre of the macula [12]. Age-related macular degeneration (AMD) was classified by the clinical Classification of Age-related Macular Degeneration method [13]. Screening for retinopathy of prematurity (ROP) was done for infants born at gestational age ≤ 32 weeks and birth weight ≤ 1500 g, and those who were given oxygen therapy in the neonatal period [14].

### Statistical analysis

**Fig. 1:**
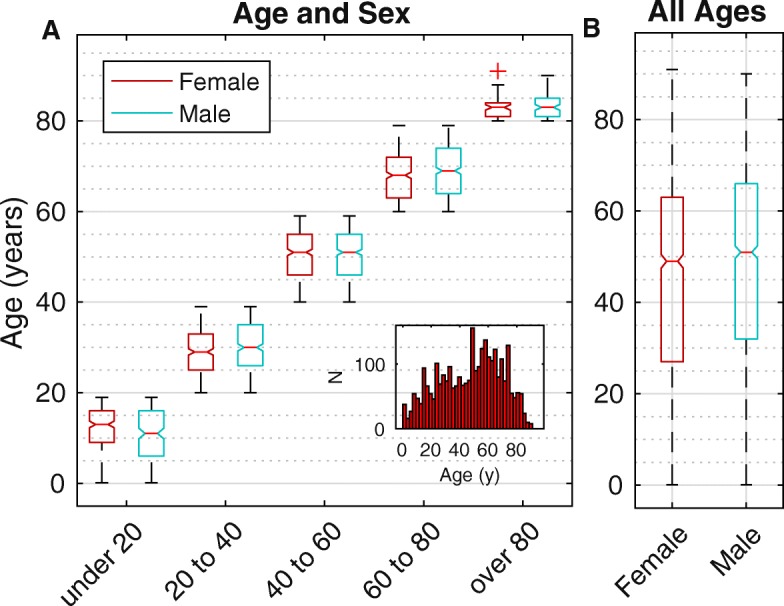
Age and Sex Boxplots. **a** Presenting ages of the 2913 patients in cohorts of 20 years. The boxes indicate 25th, 50th and 75th percentile of the age distributions. The red + represents a 91 year old female outlier. The inset shows a histogram of all the ages. A bootstrap analysis showed that overall the females presented significantly earlier than males in most cohorts. **b** In the overall distribution of ages females presented earlier than males at the 25th, 50th and 75th percentiles

The data were analysed using MATLAB (2016b, The MathWorks, Natick, MA). Comparisons of the expected and observed frequency of gender used Chi-squared tests. We applied a sampling with replacement based bootstrap analysis [15] to estimate the population means and the standard errors of the means and SE of the 10th, 25th, 50th, 75th and 90th percentiles of the ages of the males and females within 20-year cohorts (Fig. 1a), and the whole cohort (Fig. 1b). We employed 10,000 bootstrap cycles to insure the estimated means and SE converged to within 2 decimal places on 5 independent cross-validations. We then applied t-tests employing the estimated means and SE. The 2913 subjects insured that the bootstrap estimates were statistically conservative.

## Results

We saw 2913 new cases at the VRSC during the study period, and 1544 (53.0%) were males. The mean age was 47.21 ± 21.8 years (range 20 days to 91 years). Among the 1369 female patients the mean age was 45.7 ± 21.9 years with median age of 49 years, while among male patients the mean age was 48.6 ± 21.6 years with median age of 51 years. One thousand eight hundred forty-five cases (63.3%) were from urban areas, and only 1068 cases (36.7%) were from the rural settings.

The breakdown of occupation and education is given in Table 1, and the reported reasons for seeking a consultancy is given in Table 2.

**Table 1:** Breakdown of Occupation and Education distributions of patients

Occupation	Frequency	%	Education level	Frequency	Percentage
Housewife	953	32.7	Primary	570	19
Farmer	648	22.2	Graduate	363	12.5
Student	396	13.6	High School	350	12
Government employee	315	10.8	Postgraduate	86	3
Private sector employee	274	9.4	Intermediate	30	1
Religious personnel	136	4.7	Nursery	2	0.1
Retired personal	80	2.7	Did not attend modern school	1512	51.9
Corporate employee	41	1.4			
Others	70	2.4			
Total	2913	100	Total	2913	100

**Table 2:** Presenting symptoms

Presenting symptoms	Number of patients	% across symptoms
Poor vision	1222	41.9
Screening for diabetic & hypertensive retinopathy	381	13.1
Cases referred to retina clinic	282	9.7
Sudden loss of vision	271	9.3
Trauma	232	8
Vision not improving after cataract surgery	145	5
Floater	135	4.6
Headache	76	2.6
Discomfort eye	47	1.6
Flashes of light	30	1
Night blindness	23	0.8
Premature baby	13	0.4
Painful eye	11	0.4
Watering eye	9	0.3
Chloroquine treatment	6	0.2
White eye (leukocoria)	6	0.2
Others^a^	24	0.8
Total	2913	100

^a^Diplopia, proptosis, squint, black spot in visual field, foreign body sensation, Transient ischaemic attack, ENT surgery, Itching, Discharge, Fainting, Drooping eye lids

Two hundred thirty-two cases (8.0%) reported with trauma, mainly with wooden sticks, farming tools, falls, animal-related injuries, road traffic accident (RTA), domestic assaults and fights. The mode of trauma is given in Table 3. There were four cases of retained intraocular foreign body (RIOFB), one of them also had RD secondary to RIOFB.

**Table 3:** Mode of Injury

Mode	Frequency	Percentage
Road Traffic Accident	21	9.1
Fall from height	19	8.2
Assault/fight	18	7.8
Wood/stick related	14	6
Sports/Recreation related	13	5.6
Stone	5	2.2
Blast injury (bottle/firecrackers)	5	2.2
Iron rod	4	1.7
Animal related	3	1.3
Electric wire	2	0.9
Hammer/Chisel injury	2	0.9
Others	8	3.4
Unknown	118	50.8
Total	232	100

**Fig. 2:**
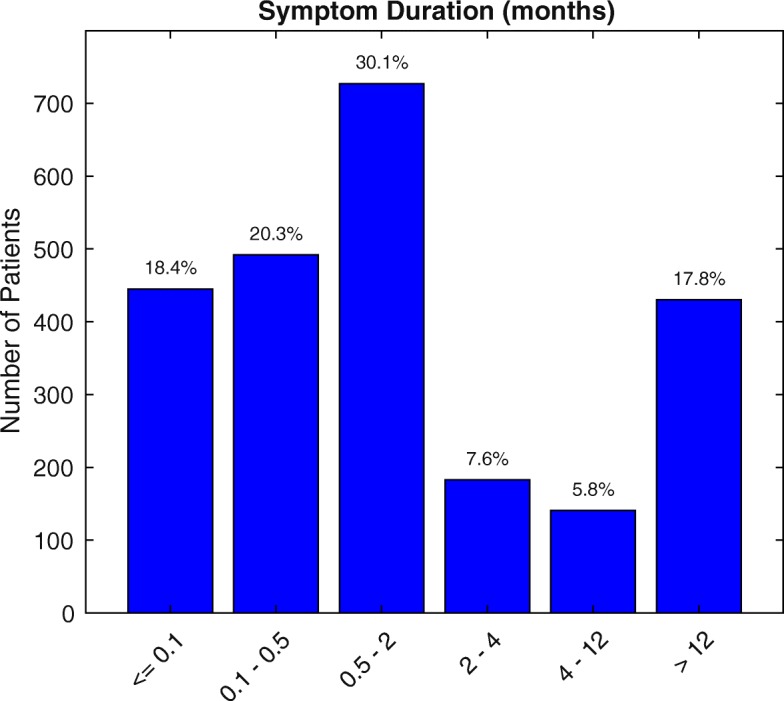
Interval between the symptom onset and presentation to the Vitreo-retinal Subspecialty Clinic (VRSC)

The interval between symptom onset and the presentation is shown in Fig. 2. Four hundred forty-five cases (18.4%) presented within 3 days of the onset of the symptoms. 492 (20.3%) presented between 0.1 to 0.5 months, 727 cases (30.1%) presented between 0.5 to 2 months, 183 cases (7.8%) presented between 2 to 4 months, 141 cases (5.8%) presented between 4 to 12 months, and 430 cases (17.8%) presented after 12 months.

**Fig. 3:**
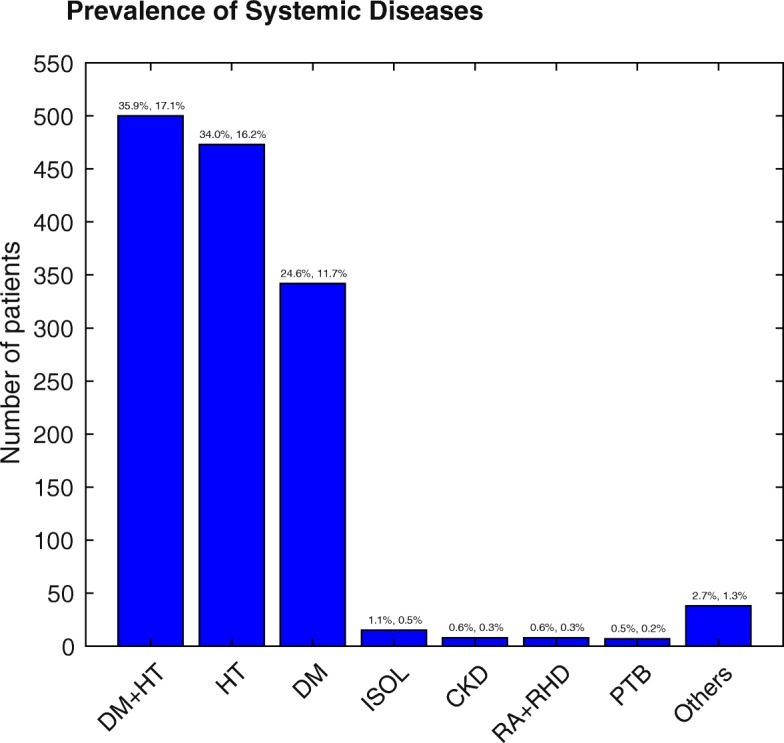
Prevalence of Systemic Diseases. Breakdown of the commonest systemic diseases found in 1380 (47.4%) of the 2913 patients. The values at the top of the columns give the percentages of the systemic disease patients (left), and the percentage of all patients (right). Others included pregnancy induced hypertension, thyroid disorders, systemic lupus erythematosus, meningitis/encephalitis, anaemia, human immunodeficiency virus, Marfans syndrome, etc

One thousand three hundred eighty cases (47.4%) had associated systemic diseases. Coexistent diabetes (DM) and hypertension (HT) was the commonest issue, found in 500 patients (35.9%), followed by HT in 473 cases (34.0%), and DM in 342 cases (24.6%) (Fig. 3). Note those percentages are of the systemic disease patients. Those percentages and those of the total population are given atop each column of Fig. 3. Brain tumours and other intracranial pathologies grouped together as intracranial space occupying lesion (ISOL) were diagnosed in 15 cases (1.1%). Chronic kidney disease (CKD) was seen in 8 patients (0.6%), and another 8 patients (0.6%) had rheumatic arthritis (RA) or rheumatoid heart disease (RHD). Seven patients (0.5%) had pulmonary tuberculosis (PTB). Other systemic diseases are detailed in Additional file 1: Table S1. The prevalence of coexistent DM and HT was 17.1% across all the patients in the study. The prevalence of other systemic diseases was 16.2% for HT, 11.7% for DM, 0.5% for ISOL, 0.3% for CKD, 0.3% for RA or RHD, 0.2% for PTB and 1.3% for other systemic diseases.

We analysed the management interventions done before the patients presented to the VRSC. Cataract surgery with intraocular lens implantation was the commonest intervention done in 146 cases (50.3% across all interventions). Retinal detachment (RD) surgery was done in 44 cases (15.1%) in India and Nepal, retinal laser in 38 cases (13.1%), corneal tear repair in 11 cases (3.8%) and brain tumour operation in 8 cases (2.8%). Other interventions are given in Additional file 2: Table S2.

**Fig. 4:**
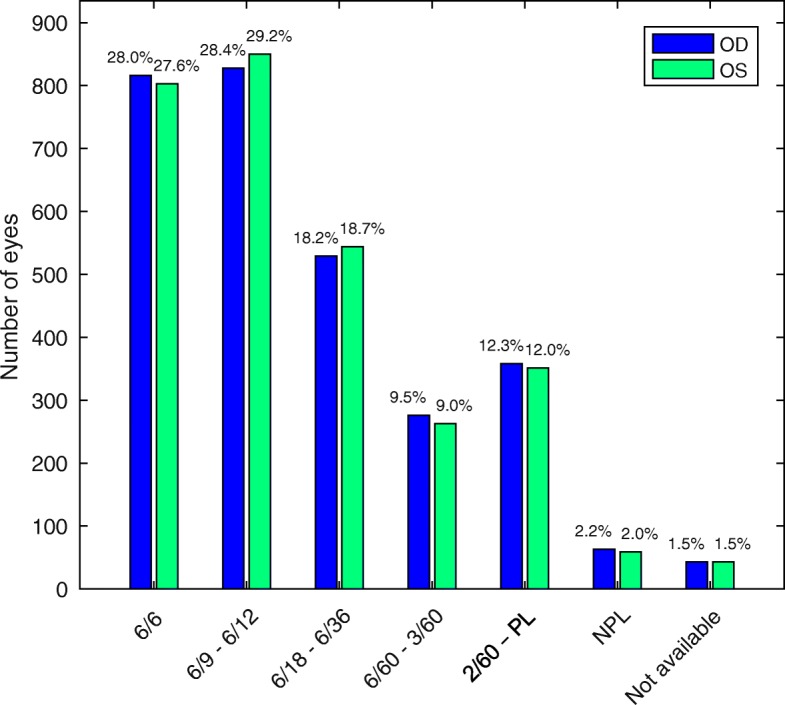
Best Corrected Visual Acuity at presentation. BCVAs between eyes were not different significantly. The BCVA data was not available in 1.5% of cases

We analysed BCVA by eye to see if the ocular dominance affected the patient presentation to the hospital, but there was none (Fig. 4). The BCVA data were not available for 41 patients who were infants and others who were not responsive.

The intraocular pressure (IOP) level ranged from hypotony (≤4 mmHg) to 74 mmHg in a case of neo-vascular glaucoma. IOP was not measured in 62 cases who were infective, toddlers in whom the measurement of IOP was not felt necessary, or patients with prosthesis or phthisis bulbi. The distribution of the IOP levels is shown in Additional file 3: Figure S1.

A diagnostic investigation was done in 2855 cases (98.0%) of total patients, while it was not felt necessary or not done in 58 cases (2.0%). Haematological test (blood sugar, glycosylated haemoglobin (HbA1c), and lipid profile) was the most common test done in 1313 cases (31.8%), followed by OCT in 1131 cases (27.4%), refraction in 410 cases (9.9%), B-scan of eye in 358 cases (8.7%), fundus photography in 328 cases (8.0%), MRI of brain and/or orbit in 155 cases (3.8%) and visual field test (Humphrey and FDT) in 144 cases (3.5%). The chest X-ray in 67 cases (1.6%) and Mantoux skin test in 66 cases (1.6%) were performed in cases of retinal vasculitis. EUA with PAC was done in 36 cases (0.9%), mainly for ROP cases. The other diagnostic tests performed were FFA, electroretinogram, vitreous culture and sensitivity, X-ray skull/orbit, CT-scan, orthoptic tests, etc. shown in Additional file 4: Table S3.

Fundus was normal in 1394 eyes and not visible in 65 eyes. These eyes were excluded for ranking the VR diseases based only on the primary diagnoses. HTR was the commonest VR disease involving 827 eyes (18.9% of all the diagnoses), followed by refractive errors referred for retinal evaluation affecting 728 eyes (16.7%), DR and CSMO in 690 eyes (15.8%), AMD in 482 eyes (11.0%), optic nerve involvement in 223 eyes (5.1%), retinal vein occlusion (RVO) in 123 eyes (2.8%), RD in 110 eyes (2.5%), glaucoma in 104 eyes (2.4%), posterior vitreous detachment (PVD) in 98 eyes (2.2%), macular scar in 88 eyes (2.0%) and macular hole in 84 eyes (1.9%). Other VR diseases are shown in Table 4.

**Table 4:** VR disease ranking based on Primary Diagnoses

Diseases	Right Eye	Left Eye	Total Eyes	% across total
Hypertensive retinopathy	409	418	827	18.9
Refractive errors referred for retinal evaluation	367	361	728	16.7
Diabetes retinopathy/CSMO	344	346	690	15.8
Age-related macular degeneration	242	240	482	11.0
Optic nerve involvement	94	129	223	5.1
Retinal vein occlusion	67	56	123	2.8
Retinal detachment	54	56	110	2.5
Glaucoma	48	56	104	2.4
Posterior vitreous detachment	48	50	98	2.2
Macular scar	51	37	88	2.0
Macular hole	49	35	84	1.9
Retinitis pigmentosa	41	42	83	1.9
Vitreous haemorrhage	39	41	80	1.8
Retinal vasculitis	30	32	62	1.4
Vitreous opacities/degeneration	29	28	57	1.3
Amblyopia	19	30	49	1.1
Retinopathy of prematurity	24	24	48	1.1
Chorioretinal scar	16	18	34	0.8
Central serous chorioretinopathy	15	19	34	0.8
Status post RD surgery	15	18	33	0.8
Hereditary maculopathy	11	11	22	0.5
Traumatic macular oedema	9	12	21	0.5
Poserior dislocated/subluxated cataract/IOL	11	8	19	0.4
Endophthalmitis	9	7	16	0.4
Panuveitis	6	10	16	0.4
Vitreo-macular traction	7	6	13	0.3
Retinal breaks	6	6	12	0.3
Others^a^	101	100	201	4.6
Total	2171	2196	4367	100

^a^Acute retinal necrosis, choroidopathy, Cystoid macular oedema, Cytomegalo virus retitinits, Chorioretinal coloboma, Central retinal artery occlusion, Coats disease, Cone dystrophy, ERM, Irido-chorio-retinal coloboma, Macular/retinal haemorrhage other than DR/AMD, myelinated nerve fibre, Nephrotic maculopathy, Open-globe injury, Optic nerve head nevus, Phthisis bulbi, Physiological cupping, Prosthesis, Retained intraocular foreign body, Localised retinal oedema/haemorrhage, Retinal telangiectasia, Retinoblastoma, Solar retinopathy, Tilted disc

We included the primary, secondary and tertiary diagnoses for the detailed description of each VR disease. A total of 840 eyes had HTR (with or without DM), of which 389 eyes (46.3%) had stage 1, 366 eyes (43.6%) had stage 2, 67 eyes (8.0%) had stage 3 and 18 eyes (2.1%) had stage 4 retinopathy. Case-wise, 253 females with mean age of 57.5 ± 14.4 years and 217 males with mean age of 55.0 ± 13.9 years had HT (*p* = 0.2). The classification DR and CSMO, and AMD is presented in Table 5. Seven hundred twenty-two eyes were affected by diabetes: 333 eyes (46.1%) had mild non-proliferative diabetic retinopathy (NPDR), 162 eyes (22.4%) had moderate NPDR, 75 eyes (10.4%) had severe and 2 eyes (0.3%) had very severe NPDR. Seventy-one eyes (9.8%) had proliferative DR (PDR) and 79 eyes (10.9%) had CSMO. Four hundred eighty-two eyes were affected by AMD: 247 eyes (51.2%) had early, 25 eyes (5.2%) had intermediate and 193 eyes (43.6%) had late AMD. Late AMD consisted of neovascular (nAMD) (139 eyes), disciform scar (49 eyes), polypoidal choroidal vasculopathy (PCV) (17 eyes) and geographic atrophy (5 eyes).

**Table 5:** Classifications of diabetic retinopathy and macular oedema and AMD

Disease	Classification	Number of eyes	% across types
Diabetes retinopathy and macular oedema	Mild NPDR	333	46.1
	Moderate NPDR	162	22.4
	Severe NPDR	75	10.4
	Very severe NPDR	2	0.3
	Proliferative DR	71	9.8
	CSMO	79	10.9
	Total	722	100
Age-related macular degeneration	Early	247	51.2
	Intermediate	25	5.2
	Late	210	43.6
	Total	482	100

Of a total of 110 RD cases, 86 cases were rhegmatogenous (RRD), 13 cases were exudative (ERD) and 11 cases were tractional (TRD). The prevalence of RD was significantly higher among females: 41 males and 83 females (*p* = 0.007).

One hundred and four eyes (2.4%) presented with glaucoma, including 48 glaucomatous OA and 3 neo-vascular glaucoma.

Eighty-eight eyes had macular scar (including 11 eyes with toxoplasmic macular scar) caused by diseases other than AMD. There were 87 cases of macular hole (MH): 76 cases (87.4%) were full-thickness MH, 9 cases (10.3%) were lamellar MH and 2 cases (2.3%) were impending MH. Sixty-two cases (89.9%) of full-thickness MH were unilateral, while 7 cases (10.1%) were bilateral. Among lamellar MH, 5 cases were unilateral and 2 cases were bilateral. Both cases of impending MH were unilateral. Sixteen cases presented with endophthalmitis, commonest being traumatic involving 7 eyes, followed by post-operative in 6 eyes and 3 endogenous. Two of the endogenous cases were diagnosed to be seasonal hyper-acute pan-uveitis (SHAPU) clinically [16].

## Discussion

We found that the majority of the patients were male. This finding agrees with other hospital-based studies on VR diseases [17, 18]. The females presented earlier than males. This might be because of the health advocacy routinely given to the women of child-bearing age in the reproductive health unit, and the eye-care education given by the eye-care professionals in the primary through to the tertiary health centres. This may also explain why housewives were the commonest group to seek consultation in the VRSC. The educational profile of the citizenry is a yardstick of development of Bhutan. Bhutan is a developing country where the majority of the patients, especially senior citizens, have not attended modern schooling. This is because the modern education was only introduced in Bhutan in the 1960s [19]. This study found that the majority of the patients were from urban areas, where educational attainment is higher and access to care is easier. Note however that the patients who came from the rural settings, but who had been living in the towns and cities for 6 months or more with their relatives, were been categorised as urban people.

Patients presenting for screening for DR and HTR were second only to poor vision as a complaint. They had either HT or DM or both. This again could be the positive impact of health advocacy. The third highest presenting complaint was referral from other health centres or inferred from other departments in the JDWNRH, reflecting the positive collaboration among the health care staff across different departments and health centres and the value of multi-disciplinary approach to health care system emphasized in the country. Sudden loss of vision was also a frequent cause for presentation. This is because of the high prevalence of HT and DM (Fig. 3) and resulting HTR, DR, CSMO, RVO and vitreous haemorrhage (VH) (Table 4). Ocular trauma was common and was mainly related to agricultural activities, domestic animals and RTA. This resulted severe ocular complications such as retained IOFB, including secondary RD [20]. This is because working with hammer and chisel, and other agricultural tools is commonly practised in Bhutan. The agriculture, livestock and forest sectors provide livelihood for about 57% of the total population [21]. The agricultural sector contributed 16.18% of the GDP of Bhutan in 2013 [21, 22]. RTA is common in Bhutan: in 2013–14 alone there were 1866 accidents resulting 1143 injuries and 157 deaths [23]. Considering the population of less than a million, this death toll due to RTA is significant [1]. Vision not improving after cataract operation was a major concern because of the patients’ dissatisfaction. The retinal examination revealed the causes of poor visual outcome were AMD, macular scar, RVO, optic atrophy (OA), DR, CSMO and cystoid macular oedema (CMO).

Eighteen percent of patients presented within 3 days from the onset of the symptoms despite that the patients outside Thimphu have to travel for a day to reach JDWNRH. They were the cases of acute conditions which cause sudden loss of vision like trauma, RVO, RD, VH, central serous chorio-retinopathy (CSCR), and endophthalmitis. Thirty percent of cases presented between 15 days to 2 months as these were the cases of acute and chronic diseases like AMD, CSMO, MH, DR, PVD, refractive errors, etc. because these conditions are not so acute, and the patients plan and decide to seek the consultation. Eighteen percent presented only after 12 months as these were the cases like HTR, DR and peripheral retinal lesions like chorioretinal scars, which are less symptomatic or asymptomatic conditions.

In our study, 47.4% of the patients had associated systemic disease. Coexistent DM and HT was the commonest (35.9%), isolated HT (34.0%) and DM (24.6%) being the second and third commonest. This finding differs from most of the other hospital-based studies on retinal diseases, which have found either HT or DM as the commonest [17, 18, 24]. The high prevalence of HT among the patients presenting to VRSC could be attributed to high altitude. The southern belt has a median altitude of 300 m, while the highest peak Gangkhar Puensum in the north is at 7570 m, and human settlement is found as high as 5250 m in Lhedi village in Lunana [25]. There is evidence that for every 100 m increase in altitude there is corresponding 2% increase in the prevalence of HT [26]. At the VRSC we also saw a spectrum of other systemic diseases (Additional file 1: Table S1). We and others have noticed that patients tend to present earlier when their dominant eye is affected [27], this most often means when their right eye is affected. Given that handedness and eye dominance are correlated [28], we analysed BCVA of right and left eyes separately (Fig. 4), but we did not find any between-eye disparity. In our similar study of VR diseases in Nepal [29] we noticed that those patients tended not to report vision problems until the acuity of their dominant eye, generally their right eye, was worse than 6/18. This resulted in a highly significant bias in the BCVA between eyes of those patients.

Blood tests for sugar, lipid profile and HbA1c were the commonest investigation done because of high prevalence of diseases with bio-markers in blood like DM, HT, RVO, VH, etc. in our study. These were also done as part of pre-operative workups. OCT was the second commonest test done (Additional file 4: Table S3) because of its utility for AMD, CSMO, RVO, CSCR, MH, macular dystrophies, CMO, myopic choroidal neovascular membrane, glaucoma, trauma, etc. OCT has become an indispensable ancillary test in the diagnosis and management of diseases involving retina and/or choroid. Substantial information is gained about the pathologic and structural changes of the ocular conditions and so OCT is commonly applied in the VRSC [30]. Refraction was third commonest test done because in our study refractive error was the second commonest disease only next to HTR. B-scans were the fourth common test done. It was applied mainly to confirm RD, VH, PVD, vitreous opacities/degenerations and in 65 cases of poor ocular media clarity. Fundus photography was done in cases of DR as baseline work up, documentation, classification using the Modified Airlie House Classification System [11], and also as a tool for evaluating the progression of the retinopathy. These were also taken in cases of RD, chorioretinal coloboma, OA, papilloedema, retinal myelinated nerve fibre, etc. The photographs also served as important tool for explaining the diseases to the patients. The visual field testing was done in VRSC because doubtful cases of glaucoma were referred to VRSC to rule out other neurological and VR diseases.

Chest X-Ray and Mantoux tests were mostly done in cases of retinal vasculitis, popularly known as Eales’ disease, to see if tuberculosis was the causative factor. Immunological, molecular biological and biochemical studies have indicated the role of human leucocyte antigen, retinal autoimmunity, *Mycobacterium tuberculosis* genome, and free radical-mediated damage in the etiopathogenesis of the disease [31]. EUA with PAC was done for screening, diagnosing and delivering laser therapies for ROP. The target infants were selected as per the ROP screening guidelines [14].

MRI of the brain and orbit was sought frequently because the cases of trauma and intracranial pathologies are referred to VRSC for ruling out raised intracranial tension, manifested clinically as papilloedema, headache, vomiting and partial or total OA [32]. Often we did MRI for confirmation or ruling out the pathology. MRI was performed in cases of optic neuritis to confirm the retrobulbar neuritis and rule out multiple sclerosis [33].

HTR was the commonest VR disease in our clinic. Partly this high prevalence of HT and HTR could be due to high altitude of Bhutan [26]. In our study 31.4% of DR had vision-threatening DR (VTDR), including severe and very severe DR, PDR and CSMO. This finding agrees with previous literature stating that a third of the DR are VTDR. It is estimated that this figure will escalate in future due to the increasing prevalence of diabetes, aging population, and growing life expectancy [34].

Our study found that 43.6% of AMD patients had late or advanced disease, whereas the literature supports that only 10% of AMD are late AMD, mainly nAMD and accounts for 90% of blindness from AMD [35]. This disparity could be due to that our study being a hospital-based and mostly the advanced AMD cases presented for treatment, and also we have included 17 eyes with PCV in late AMD group considering PCV as a subset of nAMD [36].

Interestingly, we found that the prevalence of RD was significantly higher among females (*p* = 0.007). However, the literature states that the prevalence of RD is more common among males [37–39].

We found that 87.4% of MH were full-thickness and 89.9% of these were unilateral in agreement with another study [27]. Post-traumatic endophthalmitis was the commonest type and the trauma occurred during the agricultural activities, which is the major source of income for the Bhutanese population [21, 22]. We also diagnosed three cases of endogenous endophthalmitis, two of them as SHAPU based on the clinical findings of childhood presentation, sudden onset of redness, leukocoria, profound loss of vision, minimal pain and hypotony [40].

A similar hospital-based study was conducted by some of the present authors on the pattern and presentation of VR diseases at a tertiary eye care centre in Nepal. It also reported that the majority of the patients were male (60.3% cf. 53.0% in Bhutan) [29]. Interestingly, in Nepal females presented almost a decade later than the age- and sex-matched males (*p* < 0.0001), where as in Bhutan the females presented earlier. HT was the most common systemic disease in Nepal, followed by DM. In Bhutan coexistent DM and HT was the most common, followed by HT and DM. In both the studies poor vision was the most common presenting complaint, trauma being second in Nepal but only fifth in Bhutan. In Nepal AMD was the most common VR disease, followed by DR, RVO, RP, and HTR. In Bhutan HTR was the most common VR disease. AMD ranked fourth, DR ranked third, RVO sixth, while RP ranked twelfth [29]. Population-based studies in Nepal also reported AMD was the most common VR disorder. In 2011, AMD was the most common VR disorder, followed by BRVO among the population aged ≥40 years with mean age of 70.3 ± 10.2 years [41]. In 2013, AMD was the most common VR disorder, followed by DR, HTR, macular scar, BRVO, and RD; mean age being 55.08 ± 11.51 years [7]. In 2018, AMD was the commonest VR disorder, followed by BRVO, ERM, DR, myopic degeneration, and macular hole [8].

There are some limitations in this study. JDWNRH is the single apex referral centre in Bhutan treating the vast majority of the VR diseases in the country. Therefore, the pattern of VR in this hospital may represent VR diseases in the entire citizenry. However, the difficult terrain, poor public transport and limited human resource limit the accessibility of the service. The patient registry is maintained in the out-patient department by each unit and department, but Bhutan is unique for having the patients responsible for bringing their own medical records, often in a handwritten notebook.

## Conclusions

The disease pattern reflects the economy and geographic location of a country, and the activities and life style of the population. Any action plan to control the diseases and the rehabilitation of those who are affected should be based on the disease pattern. Bhutan needs to focus on non-communicable diseases like HT and DM and their complications. There is a need to promote safer agricultural activities and stricter legislation of the traffic rules to safeguard the population. The third front is to tackle is the specific eye diseases like refractive error, AMD and glaucoma, and rare but dreadful conditions like SHAPU.

## Supplementary information

**Additional file 1: Table S1.** Systemic Diseases associated.

**Additional file 2: Table S2.** Prior Interventions done before presenting to VRSC, JDWNRH.

**Additional file 3: Figure S1.** Intraocular pressure. The readings ranged from 4 mmHg to 74 mmHg. The histogram is split into two parts to show the detail of the long tail of high IOPs above 20 mmHg corresponding the eyes above the 95th percentile of all IOPs.

**Additional file 4: Table S3.** Diagnostic Investigations.

## Data Availability

The data have not been placed in any online data storage. The datasets generated and analysed during the study are available upon request from the first author.

## Abbreviations

AMD: Age-related Macular Degeneration
BCVA: Best Corrected Visual Acuity
BRVO: Branch Retinal Vein Occlusion
CKD: Chronic Kidney Disease
CMO: Cystoid Macular Oedema
CRVO: Central Retinal Vein Occlusion
CSCR: Central Serous Chorio-retinopathy
CSMO: Clinically Significant Macular Oedema
DM: Diabetes Mellitus
DR: Diabetic Retinopathy
ERG: Electroretinogram
ETDRS: Early Treatment Diabetic Retinopathy Study
FFA: Fundus Fluorescence Angiography
HT: Hypertension
HTR: Hypertensive retinopathy
IOL: Intra-ocular Lens
IOP: Intraocular pressure
ISOL: Intracranial Space Occupying Lesions
MH: Macular Hole
nAMD: Neovascular AMD
NPDR: Non-proliferative Diabetic Retinopathy
NPL: No Perception of Light
OA: Optic Atrophy
OCT: Optical Coherence Tomography
PCV: Polypoidal Choroidal Vasculopathy
PDR: Proliferative Diabetic Retinopathy
PTB: Pulmonary Tuberculosis
PVD: Posterior Vitreous Detachment
RA: Rheumatic Arthritis
RD: Retinal Detachment
RHD: Rheumatoid Heart Disease
RIOFB: Retained Intra-ocular Foreign Body
ROP: Retinopathy Of Prematurity
RP: Retinitis Pigmentosa
RPE: Retinal Pigment Epithelium
RVO: Retinal Vein Occlusion
S/P: Status Post
SHAPU: Seasonal Hyper-Acute Pan-Uveitis
