# Supplementary material for: Pattern of vitreo-retinal diseases at the national referral hospital in Bhutan: a retrospective, hospital-based study
Source: BMC Ophthalmol. 2020 Feb 13;20:51. doi: 10.1186/s12886-020-01335-x (PMC7017569; doi:10.1186/s12886-020-01335-x)
Supplement: Supplementary file 1 — Additional file 1: Table S1. Systemic Diseases associated. [file 12886_2020_1335_MOESM1_ESM.docx]

**Table S1**. Systemic Diseases associated

|  |  |  |
| --- | --- | --- |
| **Systemic diseases** | **Disease frequency** | **% among diseases** |
|  |  |  |
| Coexistent DM & HT | 500 | 35.9 |
| Hypertension (HT) | 473 | 34 |
| Diabetes Mellitus (DM) | 342 | 24.6 |
| Brain tumour*/Epilepsy, 10+2 | 12 | 0.9 |
| Chronic Kidney Disease | 8 | 0.6 |
| Rheumatic Arthritis/RHD | 8 | 0.6 |
| Pulmonary Tuberculosis | 7 | 0.5 |
| Pregnancy-induced Hypertension | 6 | 0.4 |
| Dysthyroidism | 5 | 0.4 |
| Systemic Lupus Erythematosus | 5 | 0.4 |
| Encephalitis/Meningitis | 3 | 0.2 |
| Anaemia | 3 | 0.2 |
| HIV infection | 2 | 0.1 |
| Marfans syndrome | 2 | 0.1 |
| Others** | 12 | 0.9 |
| **Total systemic diseases** | **1388** | **100** |
| Patients without systemic diseases | 1533 | 52.6 |
| Patients with systemic disease | 1380 | 47.4 |
| **Total patients in the study** | **2913** | **100** |

| *Tumour, craniopharyngioma, Empty sella syndrome, hydrocephalus, pituitary cyst and macroadenoma. |
| --- |
| **Carcinoma lung, Congestive cardiac failure, Chronic obstructive pulmonary disease,  Cerebrovascular accident, Downs syndrome, Deep vein thrombosis, Hepatitis B, Pheochromocytoma,  Masquerade syndrome, Myesthenia gravis, Non-Hodgkin’s lymphoma, Syphilis. |
