# Supplementary material for: Pattern of vitreo-retinal diseases at the national referral hospital in Bhutan: a retrospective, hospital-based study
Source: BMC Ophthalmol. 2020 Feb 13;20:51. doi: 10.1186/s12886-020-01335-x (PMC7017569; doi:10.1186/s12886-020-01335-x)
Supplement: Supplementary file 2 — Additional file 2: Table S2. Prior Interventions done before presenting to VRSC, JDWNRH. [file 12886_2020_1335_MOESM2_ESM.docx]

| **Table S2**. Prior Interventions done before presenting to VRSC, JDWNRH | | | |
| --- | --- | --- | --- |
|  | | |  |
| **Interventions** | **Number of eyes** | **% across interventions** |  |
|  | | |  |
| Cataract operation + IOL | 146 | 49.2 |  |
| Retinal detachment surgery | 44 | 14.8 |  |
| Retinal laser | 37 | 12.5 |  |
| Corneal tear repair | 15 | 5.1 |  |
| Brain tumor operation | 8 | 2.7 |  |
| Scleral tear repair | 8 | 2.7 |  |
| Lensectomy | 5 | 1.7 |  |
| Lid Tear Repair | 5 | 1.7 |  |
| Pterygium excision with graft | 5 | 1.7 |  |
| Anti-VEGF injection | 4 | 1.4 |  |
| Cancer Chemotherapy | 3 | 1.0 |  |
| Trabeculectomy | 3 | 1.0 |  |
| Pars plana vitrectomy | 2 | 0.7 |  |
| Renal transplant | 2 | 0.7 |  |
| Others* | 10 | 3.4 |  |
| **Total interventions done** | **297** | **100** |  |
| No. of patients without intervention | 2627 | 90.2 |  |
| No. of patients with intervention | 286 | 9.8 |  |
| **Total patients** | **2913** | **100** |  |

*Conjunctival tear repair, Cryotherapy, Hemodialysis, Injection methyl prednesolone, Injection Vancomycin + Ceftazidime + Dexamethasone, Laser peripheral iridotomy, Lasik, Macular hole surgery, Parotid gland operation, Radiotherapy.
