# Supplementary figures and images for: Pattern of vitreo-retinal diseases at the national referral hospital in Bhutan: a retrospective, hospital-based study
Source: BMC Ophthalmol. 2020 Feb 13;20:51. doi: 10.1186/s12886-020-01335-x (PMC7017569; doi:10.1186/s12886-020-01335-x)

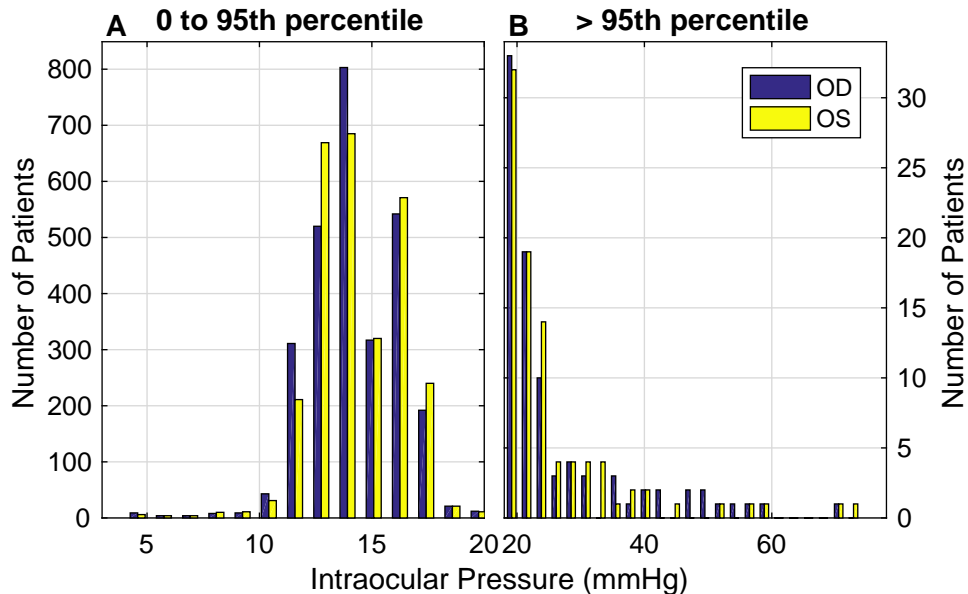

Supplement: Supplementary file 3 — Additional file 3: Figure S1. Intraocular pressure. The readings ranged from 4 mmHg to 74 mmHg. The histogram is split into two parts to show the detail of the long tail of high IOPs above 20 mmHg corresponding the eyes above the 95th percentile of all IOPs. [file 12886_2020_1335_MOESM3_ESM.pdf]
