# Supplementary material for: Pattern of vitreo-retinal diseases at the national referral hospital in Bhutan: a retrospective, hospital-based study
Source: BMC Ophthalmol. 2020 Feb 13;20:51. doi: 10.1186/s12886-020-01335-x (PMC7017569; doi:10.1186/s12886-020-01335-x)
Supplement: Supplementary file 4 — Additional file 4: Table S3. Diagnostic Investigations. [file 12886_2020_1335_MOESM4_ESM.docx]

| **Table S3. Diagnostic Investigations** | | |
| --- | --- | --- |
| **Tests** | **Test frequency** | **% across tests** |
|  |  |  |
| *Haematological tests | 1,313 | 31.8 |
| Optical Coherence Tomography | 1,131 | 27.4 |
| Refraction | 411 | 9.9 |
| B-scan | 358 | 8.7 |
| Fundus photography | 331 | 8.0 |
| MRI Brain/Orbit | 155 | 3.8 |
| Visual Field (HVF+FDT) | 144 | 3.5 |
| Chest X-Ray | 67 | 1.6 |
| Mantoux test | 66 | 1.6 |
| Examination Under Anaesthesia | 36 | 0.9 |
| X-ray skull | 26 | 0.6 |
| Fundus Fluorescein Angiography | 23 | 0.6 |
| CT-scan | 22 | 0.5 |
| Vitreous culture/sensitivity | 16 | 0.4 |
| Orthoptic test | 14 | 0.3 |
| Electroretinogram | 12 | 0.3 |
| Others** | 7 | 0.2 |
| **Total tests done** | **4,132** | **100** |
| No. of patients with diagnostic tests done | 2,855 | 98.0 |
| No. of patients without any test | 58 | 2.0 |
| **Total patients** | **2,913** | **100** |
|  |  |  |

*Blood sugar, lipid profile & HbA1c; **Cytology, Diplopia charting, Lacrimal syringing
